# Noncontact Sleep Monitoring With Infrared Video Data to Estimate Sleep Apnea Severity and Distinguish Between Positional and Nonpositional Sleep Apnea: Model Development and Experimental Validation

**DOI:** 10.2196/26524

**Published:** 2021-11-01

**Authors:** Sina Akbarian, Nasim Montazeri Ghahjaverestan, Azadeh Yadollahi, Babak Taati

**Affiliations:** 1 Kite Research Institute Toronto Rehabilitation Institute University Health Network Toronto, ON Canada; 2 Institute of Biomaterials and Biomedical Engineering University of Toronto Toronto, ON Canada; 3 Vector Institute Toronto, ON Canada; 4 Department of Computer Science University of Toronto Toronto, ON Canada

**Keywords:** sleep apnea, deep learning, noncontact monitoring, computer vision, positional sleep apnea, 3D convolutional neural network, 3D-CNN

## Abstract

**Background:**

Sleep apnea is a respiratory disorder characterized by frequent breathing cessation during sleep. Sleep apnea severity is determined by the apnea-hypopnea index (AHI), which is the hourly rate of respiratory events. In positional sleep apnea, the AHI is higher in the supine sleeping position than it is in other sleeping positions. Positional therapy is a behavioral strategy (eg, wearing an item to encourage sleeping toward the lateral position) to treat positional apnea. The gold standard of diagnosing sleep apnea and whether or not it is positional is polysomnography; however, this test is inconvenient, expensive, and has a long waiting list.

**Objective:**

The objective of this study was to develop and evaluate a noncontact method to estimate sleep apnea severity and to distinguish positional versus nonpositional sleep apnea.

**Methods:**

A noncontact deep-learning algorithm was developed to analyze infrared video of sleep for estimating AHI and to distinguish patients with positional vs nonpositional sleep apnea. Specifically, a 3D convolutional neural network (CNN) architecture was used to process movements extracted by optical flow to detect respiratory events. Positional sleep apnea patients were subsequently identified by combining the AHI information provided by the 3D-CNN model with the sleeping position (supine vs lateral) detected via a previously developed CNN model.

**Results:**

The algorithm was validated on data of 41 participants, including 26 men and 15 women with a mean age of 53 (SD 13) years, BMI of 30 (SD 7), AHI of 27 (SD 31) events/hour, and sleep duration of 5 (SD 1) hours; 20 participants had positional sleep apnea, 15 participants had nonpositional sleep apnea, and the positional status could not be discriminated for the remaining 6 participants. AHI values estimated by the 3D-CNN model correlated strongly and significantly with the gold standard (Spearman correlation coefficient 0.79, *P*<.001). Individuals with positional sleep apnea (based on an AHI threshold of 15) were identified with 83% accuracy and an F1-score of 86%.

**Conclusions:**

This study demonstrates the possibility of using a camera-based method for developing an accessible and easy-to-use device for screening sleep apnea at home, which can be provided in the form of a tablet or smartphone app.

## Introduction

**Figure 1:**
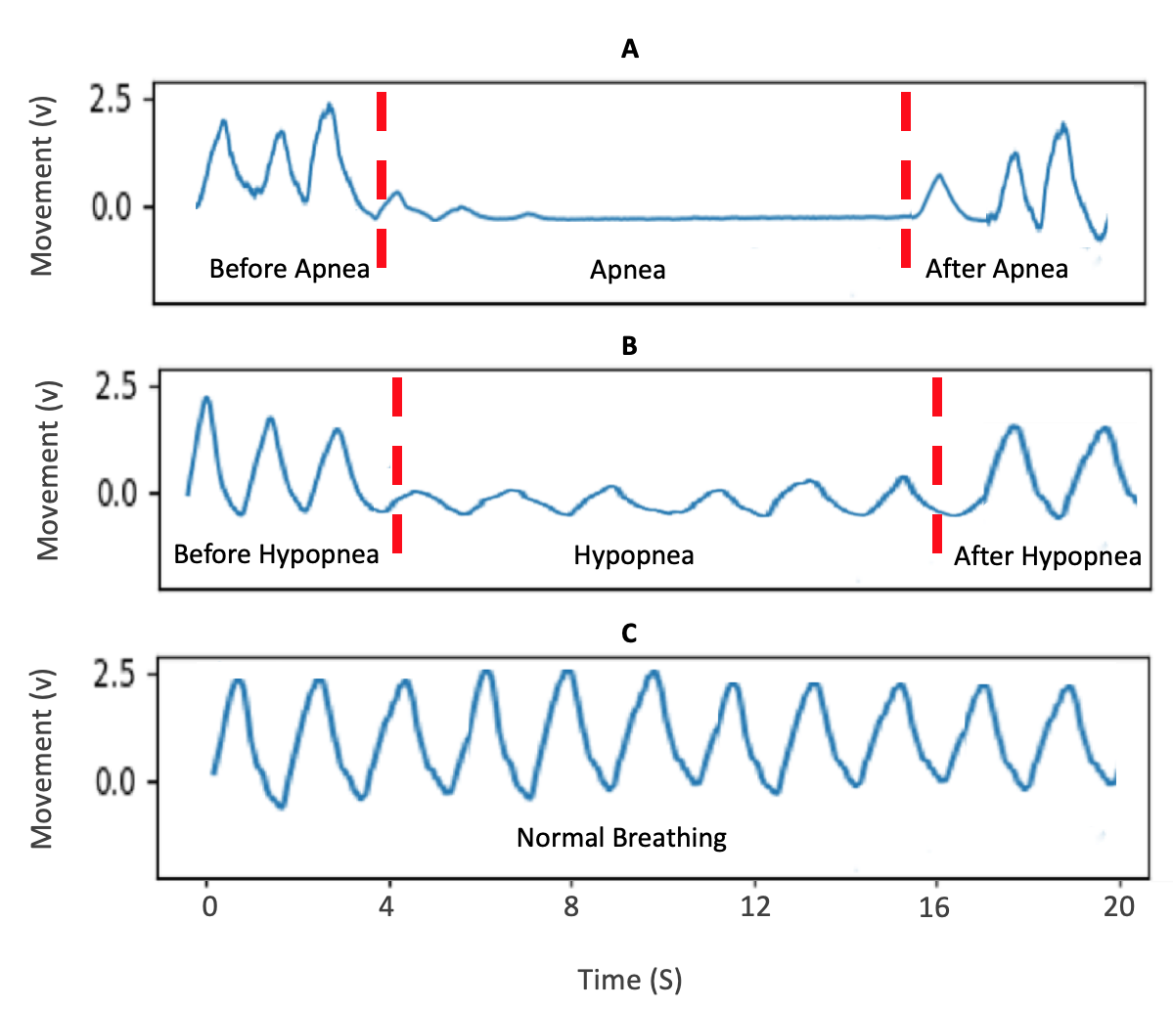
Sample sum of chest and abdomen movements in (A) apnea, (B) hypopnea, and (C) normal breathing.

Sleep apnea is a chronic respiratory disorder occurring due to frequent respiratory airflow reduction during sleep. Cessation of airflow lasting for more than 10 seconds is called apnea, whereas partial reduction in airflow by more than 30% for at least 10 seconds—in association with more than a 3% drop in blood oxygen saturation level or arousals—is called hypopnea. Sample images indicating the chest movements during normal breathing, hypopnea, and apnea are shown in Figure 1. The apnea-hypopnea index (AHI) is an indicator of the severity of sleep apnea, which measures the hourly occurrence rate of apneas and hypopneas [1]. Untreated sleep apnea raises the risk of hypertension, heart diseases, and stroke [2].

Positional sleep apnea refers to sleep apnea patients for whom the AHI in the supine sleeping position is at least 50% higher than that in the nonsupine sleeping positions [3]. Recent studies have shown that changing to a lateral sleeping position can decrease the AHI for patients with positional sleep apnea [4]. This behavioral intervention is known as “positional therapy,” and is an effective noninvasive and nonpharmaceutical treatment for those with positional sleep apnea [5].

The current clinical approach to diagnose sleep apnea and to determine whether or not it is positional is based on polysomnography (PSG). However, PSG requires connecting more than 20 sensors to a user, which is inconvenient. A trained sleep technician manually analyzes recorded PSG signals and annotates the sleep position overnight. Moreover, PSG is expensive (>US $400) and has a long waiting time in some areas (4-36 months in Canada [6]). As a result, up to 85% of the population at risk of sleep apnea remain undiagnosed [7]. It is therefore useful to investigate screening technologies that could identify individuals at high risk via a simpler test. Increasing access to testing, diagnosis, and subsequent treatment could improve the patient’s quality of life by decreasing hypertension and sleepiness, and can also reduce overall health care costs [8-10].

Researchers have developed several easy-to-use, convenient, and accessible methods for sleep apnea monitoring. Merchant et al [11] developed a skin-adhesive patch recording nasal pressure, blood oxygen saturation, pulse rate, respiratory effort, sleep time, and body position to estimate the AHI. Ayas et al [12] evaluated the performance of a wrist-worn device utilizing a peripheral arterial tonometer, actigraphy, and arterial oxygen saturation to diagnose sleep apnea. Varon et al [13] introduced a method for the automatic detection of sleep apnea from single-lead electrocardiogram by training a least-squares support vector machines classifier on the features extracted from the electrocardiogram signal. Several studies estimated AHI and respiratory events from analyzing tracheal sound or tracheal movements, or the combination of tracheal sound with oxygen saturation [14-18]. Lévy et al [19] utilized pulse oximetry to quantify arterial oxygen saturation and to diagnose sleep apnea.

Although these methods are more convenient than PSG, sensors attached to the body could potentially disrupt the user’s regular sleep pattern. Therefore, researchers have continued to develop noncontact methods to screen individuals at risk of sleep apnea. For example, we previously developed a deep-learning model to distinguish between different types of apnea. However, as the model was not capable of detecting events, we used ground truth labels for this purpose [20]. Jakkaew et al [21] used a thermal camera to estimate breathing rate and body movements; however, they did not analyze the breathing pattern to identify sleep apnea, and the method was not designed to detect sleep position. Deng et al [22] used six active infrared cameras and a Kinect sensor to detect body position and breathing pattern (abnormal vs normal breathing). However, they did not evaluate their method in a clinical environment to demonstrate the performance for the detection of sleep apnea or positional sleep apnea. In addition, using six cameras and the Kinect will be difficult to set up in clinical or home settings, which hinders large-scale adoption. Davidovich et al [23] developed a new framework to extract the breathing pattern from a piezo-electric sensor placed under the patient’s mattress through extracting time and frequency domain features and then calculating the AHI. Nandakumar et al [24] used a smartphone to emit inaudible waves and to analyze the waves’ echoes from the user’s body to detect respiratory events. However, these noncontact methods did not present cross-validation performance, and due to restriction in their modalities, they are not able to identify positional sleep apnea patients, which is crucial for proper treatment.

To identify patients at risk of sleep apnea and to distinguish those with positional sleep apnea, an alternative is to use computer vision and machine-learning techniques. We here propose a noncontact algorithm that analyzes infrared videos captured from a participant during sleep to estimate the AHI and to distinguish patients with positional vs nonpositional sleep apnea. Specifically, we used a 3D convolutional neural network (CNN) to analyze movements in infrared videos, to detect apneas, and to estimate the AHI. In experimental evaluation, this model outperformed a baseline model that previously reported state-of-the-art results in noncontact AHI estimation [25]. We also combined this technique with another CNN-based approach that detects the sleeping position [26] to calculate the AHI in different sleeping positions and to identify patients with positional sleep apnea. The methods and results developed in this study represent the first noncontact approach to automatically distinguish positional from nonpositional sleep apnea.

## Methods

### Data Collection

The University Health Network Research Ethics Board approved this study (approval number 13-7210-DE). Participants aged 18 to 85 years and without a history of cardiovascular or renal diseases were recruited for this study. Participants were recruited among patients referred for sleep diagnosis at the sleep laboratory of the Toronto Rehabilitation Institute, University Health Network. All participants signed a written consent form before taking part in the study. There were no limitations on blanket usage, movement, or clothing worn during sleep.

Simultaneously with overnight PSG (Embla s4500) that was used for a clinical diagnosis of sleep, infrared videos of participants were recorded at a resolution of 640×480 with 30 frames per second. The participants’ video data were collected and synchronized with PSG signals all night for 5 (±1) hours while sleeping in a single session.

**Figure 2:**
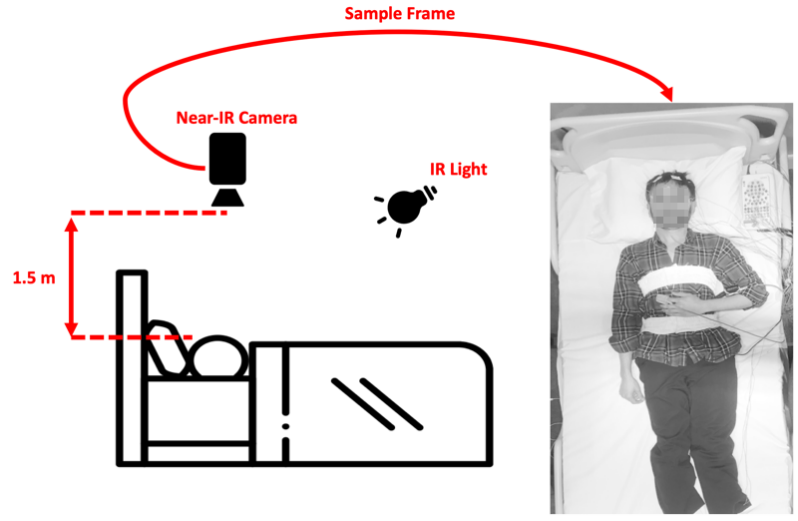
Data collection setup and a sample anonymized image frame on the right. IR: infrared.

The infrared camera (Point Grey Firefly MV, 0.3 MP, FMVU-03MTM) was mounted approximately 1.5 meters above the bed. For illumination, a separate infrared light source (Raytec RM25-F-50) was mounted on the ceiling. A schematic of the camera setup and sample frame is shown in Figure 2.

Respiratory events (apneas and hypopneas) and sleep positions (supine, lateral) of the participant throughout the night were annotated by a trained sleep technician who was blinded to the study. Since the video data were synchronized with PSG data, once the technician annotated the PSG data, all video frames were automatically labeled.

### AHI Estimation

**Figure 3:**
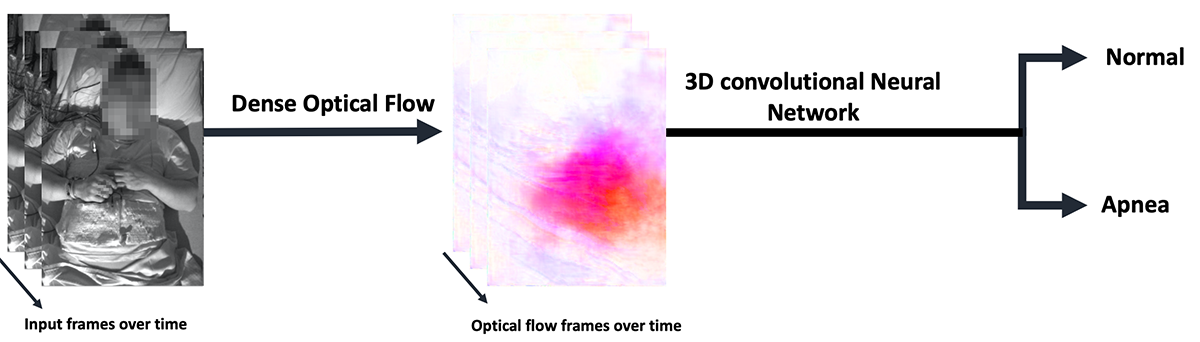
Sample input and dense optical flow images.

The video frames were first downsampled from 30 Hz to 2 Hz to reduce the computational cost. As breathing frequency is approximately 0.5 Hz during sleep, the reduced frequency of 2 Hz exceeds the Nyquist rate by a factor of 2. To track respiratory movements in the infrared video frames, a CNN dense optical flow (Flownet 2.0 [27]) was used, which provides accurate optical flow at a fast frame rate. Optical flow extracts movement in the *x* (side to side) and *y* (up and down) directions for each pixel in one video frame to the next. The minimum duration of an apnea is 10 seconds. This translates to 20 (or 19 in the worst case) video frames within the duration of an event. To estimate respiratory events, a 3D-CNN was trained on a sliding window of 18 optical flow images (ie, resulting from 19 consecutive video frames). Infrared videos were captured at a resolution of 640×480 pixels, resulting in optical images with a size of 640×480×2. The architecture of the 3D-CNN that was trained on the input tensors with a size of 640×480×2×18 is shown in Multimedia Appendix 1. Sample input and dense optical flow images are shown in Figure 3.

The 3D-CNN was trained with class-weighted cross-entropy loss (5 for events and 1 for normal) and the Adam optimizer. An initial value of 0.001 for the learning rate and a batch size of 25 for 25,000 epochs were chosen. The total number of parameters in this network was 8,284,265, including 8,281,829 trainable parameters and 2436 nontrainable parameters. Depending on the sleep apnea severity, respiratory events are less frequent in comparison to normal breathing; thus, the data sets were highly imbalanced. In training time, to balance the data set, stride lengths of 0.5 and 15 seconds were used for apneas and normal breathing, respectively. In test time, a stride length of 0.5 seconds was used to predict the binary label of normal breathing versus apneas. The threshold of the trained binary classification (event vs normal) was set to 0.1 to maximize the area under the curve on the training data.

To estimate the AHI, a linear regression model was trained on the following three features: (1) the number of detected events, (2) the total duration of detected events longer than 9 seconds divided by sleep duration, and (3) sleep duration.

The performance of the 3D-CNN was compared against another approach developed by our group, which previously demonstrated state-of-the-art performance in noncontact vision-based estimation of the AHI [25]. A brief overview of this baseline approach is presented here. To extract respiratory-related motion, movements of 768 uniformly scattered points in the video frames were extracted using a sparse optical flow. Principal component analysis (PCA) was applied on the extracted point trajectories over 30-second sliding windows with a stride of 1 second to compute the predominant movements, which were associated with breathing during sleep [28]. This approach was previously validated by Zhu et al [29] and was shown to accurately track breathing rate in overnight infrared videos. To identify respiratory events from the respiratory-related motion, three features were extracted, including the respiratory rate, average power of respiratory movement, and total displacement of tracked points. Compared to normal breathing, the respiratory rate drops during respiratory events. To extract the respiratory rate, the energy of extracted respiratory movements was calculated using fast Fourier transform with a window of 10 seconds. The frequency associated with the highest energy was then considered as the respiratory rate. The second feature was the average power of respiratory movement, which decreases during a respiratory event. This feature was computed as the mean of absolute squares of respiratory displacement within a 10-second window. The last feature was total displacement, which indicates nonrespiratory movement (eg, arousals), and was determined by the summation of all of the raw optical flow movements (before applying PCA). Using these 3 features, a random forest binary classifier with 50 trees was trained to estimate sleep apnea events (apneas and hypopneas). Finally, to estimate the AHI, a linear regression model was trained using 2 features: (1) the number of predicted sleep apnea events normalized by the estimated events’ duration and (2) the estimated events’ duration normalized by the total sleep duration obtained from the total recording time.

### Detecting Positional vs Nonpositional Sleep Apnea

**Figure 4:**
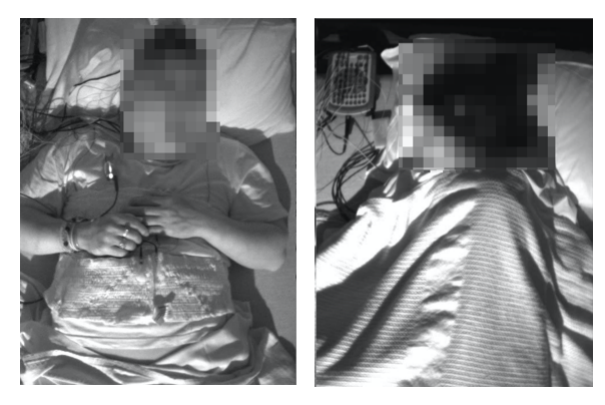
Sample supine (left) and lateral (right) frames.

For sleep position detection, a previously developed algorithm [26] was used. This method estimates body position (supine vs lateral) from a video frame using a CNN. Sample supine and lateral images are shown in Figure 4. This position detector was applied to the first video frame of each video. After each large movement (detected by thresholding the total displacement of tracked featured points extracted by optical flow over 1 second), the detector was used again to estimate the new sleeping position. As a result, a body position (supine vs lateral) was assigned to each video frame during the entire sleeping period. Once respiratory events and their associated sleep positions were detected, 6 features were calculated per person: (1) number of detected events in supine position, (2) number of detected events in lateral position, (3) total recording time in supine position, (4) total recording time in lateral position, (5) supine AHI, and (6) lateral AHI. These features were then used to train a binary random forest classifier with three trees to distinguish between positional and nonpositional sleep apnea patients.

### Validation

Leave-one-person-out cross-validation was used to evaluate the performance of AHI estimation as well as the performance of positional vs nonpositional sleep apnea detection algorithms. Bland-Altman plots and Spearman correlation coefficients were used to evaluate the performance of AHI estimation. Since an AHI of 15 is commonly used as a threshold for screening sleep apnea [30], the algorithm performance on classifying subjects as having sleep apnea was evaluated based on the threshold of AHI=15. Confusion matrices, accuracy, precision, recall, and F1-score measures were used to assess classification performance. The same measures were used to assess the performance of positional vs nonpositional sleep apnea classification.

## Results

Demographic information of the 41 individuals (26 men and 15 women) recruited for this study is shown in Table 1. There were 20 participants with positional sleep apnea, 15 participants with nonpositional sleep apnea, and 6 participants that only slept in one position and as such the apnea could not be identified as either positional or nonpositional.

**Table 1:** Participants’ demographic features for apnea-hypopnea index (AHI) estimation (N=41).^a^

Characteristics	Value, mean (SD)
Age (years)	53 (13)
BMI (kg/m^2^)	30 (7)
Sleep duration (hours)	5 (1)
Number of changes in body position	9 (6)
Sleep efficiency (%)	75 (18)
REM^b^ sleep percentage (%)	15 (7)
Mean wake heart rate (bpm^c^)	68 (16)
Mean REM heart rate (bpm)	67 (16)
Minimum SaO_2_^d^	82 (9)
Mean SaO_2_	94 (3)
AHI (events/hour)	27 (31)
Supine AHI (events/hour)	41 (39)
Lateral AHI (events/hour)	21 (34)

^a^Participants’ information was obtained from the sleep reports of the overnight sleep study annotated by sleep technicians.

^b^REM: rapid eye movement.

^c^bpm: beats per minute.

^d^SaO_2_: arterial oxygen saturation.

The threshold used in this study for detecting position changes and ignoring the small movements (eg, breathing or pulse) was empirically set to 20,000 pixels. The total displacement was calculated by summing the displacement of all optical flow feature points [28] over 1 second and was checked against this threshold.

**Figure 5:**
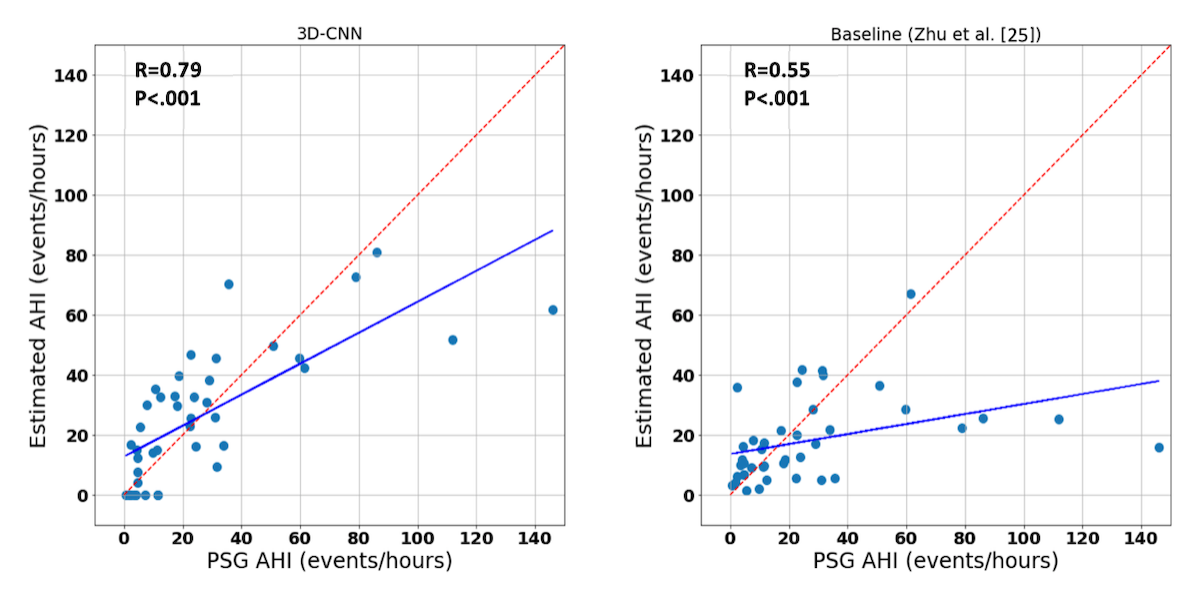
Scatterplots of polysomnography (PSG) apnea-hypopnea index (AHI) vs estimated AHI values. The blue and red lines indicate fitted and unity lines, respectively. CNN: convolutional neural network.

**Figure 6:**
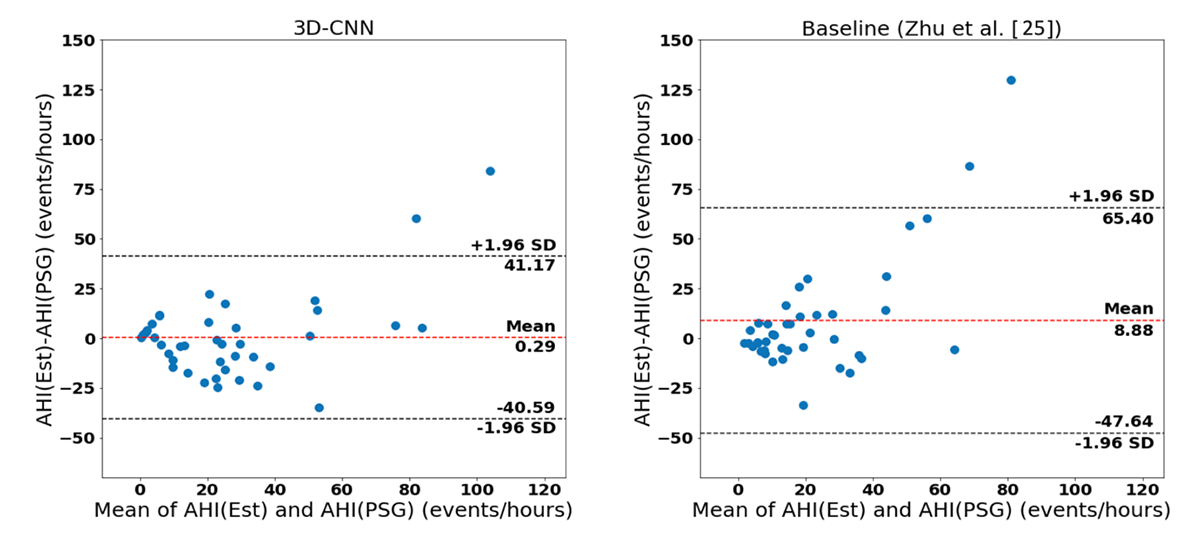
Bland-Altman plots of apnea-hypopnea index (AHI) estimation algorithms. PSG: polysomnography; Est: estimated; CNN: convolutional neural network.

To evaluate the performance of AHI detection, Figure 5 and Figure 6 show the scatterplots and Bland-Altman plots between the estimated AHI and PSG-based AHI for both the 3D-CNN model and the baseline model (Zhu et al [25]).

**Figure 7:**
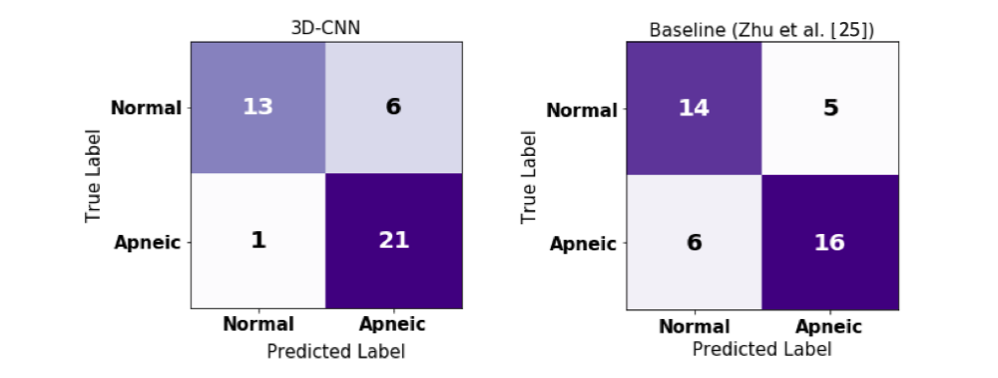
Confusion matrices for screening patients with sleep apnea based on the apnea-hypopnea index threshold of 15. CNN: convolutional neural network.

The Spearman correlation coefficients (ρ) for AHI estimation were 0.55 and 0.79 for the baseline and 3D-CNN approach, respectively (*P*<.001 in both cases). In addition, the Bland-Altman plot indicated that our method outperformed the baseline according to the smaller mean (0.3 vs 8.9) and tighter 95% limits of agreement (ie, a smaller value for 1.96 of the standard deviation: 40.9 vs 56.5). Confusion matrices and the performance measures for identifying sleep apnea patients based on the AHI=15 threshold are shown in Figure 7 and in Table 2, respectively. The 3D-CNN approach obtained 83% accuracy and an F1-score of 86%, outperforming the baseline approach, which obtained an accuracy of 73% and an F1-score of 74%.

**Table 2:** Performance of models on screening patients with sleep apnea.

Method	Accuracy	Precision	Recall	F1-score
3D-CNN^a^	82.93	77.78	95.45	85.71
Baseline (Zhu et al [25])	73.17	76.19	72.73	74.42

^a^CNN: convolutional neural network.

**Figure 8:**
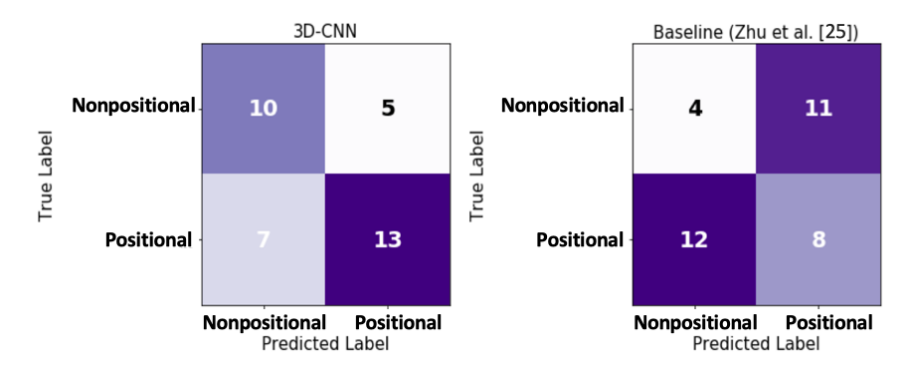
Confusion matrix for identifying positional sleep apnea. CNN: convolutional neural network.

The position detection algorithm estimated the body position with 83% accuracy, an F1-score of 83%, 77% precision, and 91% recall. The performance of the combination of the position detection algorithm with AHI detection on patients with positional sleep is shown in Figure 8. The 3D-CNN model classified 13 out of 20 patients with positional sleep apnea correctly. Performance measures for detecting positional vs nonpositional sleep apnea are presented in Table 3.

**Table 3:** Performance of the models in detecting positional vs nonpositional sleep apnea.

Method	Accuracy	Precision	Recall	F1-score
3D-CNN^a^	65.71	72.22	65.00	68.42
Baseline (Zhu et al [25])	34.29	42.11	40.00	41.03

^a^CNN: convolutional neural network.

## Discussion

### Principal Findings

The main contributions of this study are: (1) the development and experimental validation of a new noncontact approach to estimate AHI, and (2) application of this method to automatically identify individuals with positional sleep apnea. The newly developed 3D-CNN–based method outperformed the baseline model in estimating the AHI in infrared video data. However, it was ~4 times slower than the baseline algorithm. Nevertheless, the new model could still process 5 hours of sleep data in ~20 hours. Through combining estimated sleeping position information with estimated AHI, this is the first noncontact method that can identify a positional sleep apnea patient.

The developed algorithm achieved comparable performance to existing contact methods (eg, those using a single wearable sensor or a sensor placed under the mattress). For example, Hafezi et al [15] analyzed tracheal movements captured by an accelerometer to estimate AHI and to identify patients with sleep apnea. They reported a Spearman correlation of 0.86 between estimated and ground-truth (PSG) AHI values, and accuracy and F1-score values of 84% and 82%, respectively, in detecting individuals with AHI≥15. As such, they achieved a higher correlation coefficient (0.86 vs 0.79) but a lower F1-score (82% vs 86%) than our noncontact approach. An advantage of using a noncontact method over contact-based approaches is ease of use and convenience. Davidovich et al [23] used a piezo-electric sensor under a mattress to estimate the AHI. They obtained an R^2^ value of 0.86 for AHI estimation, and accuracy and F1-score values of 88% and 84%, respectively, in identifying individuals with AHI≥15. Using a camera has the potential to result in a more accessible assessment technology, as it can be implemented in the form of a tablet or mobile phone app.

### Limitations

Our study has some limitations. One limitation is the failure of the event detection algorithm when the participant moved out of the field of view of the camera or when the room lighting condition suddenly changed. Another limitation is the small number of participants (N=41). The algorithm was validated via leave-one-person-out cross-validation. Future work should examine the generalizability of these models to data collected in new environments.

### Conclusion and Future Work

This study applied machine learning and computer vision approaches to develop a CNN-based method to detect respiratory events in different sleeping positions from data collected via an infrared camera. This method was validated on data from 41 participants to estimate AHI and to identify patients with positional sleep apnea.

This model could be used toward the development of affordable and easy-to-use technologies for screening sleep apnea at home (eg, in the form of a tablet or smartphone app). Such a system could help physicians in choosing suitable treatments for sleep apnea patients. Ultimately, improved treatment will reduce the consequences of untreated sleep apnea such as car accidents, heart disease, diabetes, and high blood pressure.

## Appendix

Multimedia Appendix 1 Architecture of a 3D convolutional neural network used to detect apneas.

## Abbreviations

AHI: apnea-hypopnea index
CNN: convolutional neural network
PCA: principal component analysis
PSG: polysomnography
