# Supplementary material for: Noncontact Sleep Monitoring With Infrared Video Data to Estimate Sleep Apnea Severity and Distinguish Between Positional and Nonpositional Sleep Apnea: Model Development and Experimental Validation
Source: J Med Internet Res. 2021 Nov 1;23(11):e26524. doi: 10.2196/26524 (PMC8593819; doi:10.2196/26524)
Supplement: Multimedia Appendix 1 [file jmir_v23i11e26524_app1.docx]

Architecture of a 3D convolutional neural network used to detect apneas.

| Layer | Number of filters, n | Size/stride | Activation function | Output size |
| --- | --- | --- | --- | --- |
| Input | N/A^a^ | N/A | N/A | 480×640×18×2 |
| Average pool | N/A | 20×20×1/10×10×1 | N/A | 47×63×18×2 |
| Batch normalization | N/A | N/A | N/A | 47×63×18×2 |
| Convolutional | 64 | 5×5×1/1×1×1 | N/A | 43×59×18×64 |
| Batch normalization | N/A | N/A | Leaky Relu^b^ | 43×59×18×64 |
| Dropout | N/A | N/A | N/A | 43×59×18×64 |
| Convolutional | 64 | 10×10×1/1×1×1 | N/A | 34×50×18×64 |
| Batch normalization | N/A | N/A | Leaky Relu | 34×50×18×64 |
| Convolutional | 128 | 5×5×2/1×1×1 | N/A | 30×46×17×128 |
| Batch normalization | N/A | N/A | Leaky Relu | 30×46×17×128 |
| Convolutional | 256 | 5×5×1/1×1×1 | N/A | 26×42×17×256 |
| Batch normalization | N/A | N/A | Relu | 26×42×17×256 |
| Dropout | N/A | N/A | N/A | 26×42×17×256 |
| Max pool | N/A | 10×10×1/1×1×1 | N/A | 17×33×17×256 |
| Convolutional | 256 | 5×5×2/1×1×1 | N/A | 13×29×16×256 |
| Batch Normalization | N/A | N/A | Relu | 13×29×16×256 |
| Convolutional | 128 | 5×5×1/1×1×1 | N/A | 9×25×16×128 |
| Batch Normalization | N/A | N/A | Relu | 9×25×16×128 |
| Convolutional | 64 | 2×2×2/1×1×1 | N/A | 8×24×15×128 |
| Batch Normalization | N/A | N/A | Relu | 8×24×15×128 |
| Convolutional | 128 | 3×3×2/1×1×1 | N/A | 6×22×14×128 |
| Batch Normalization | N/A | N/A | Relu | 6×22×14×128 |
| Convolutional | 64 | 5×5×2/1×1×1 | N/A | 2×18×13×64 |
| Batch Normalization | N/A | N/A | Relu | 2×18×13×64 |
| Flatten | N/A | N/A | N/A | 29,952 |
| Fully connected | 64 | 29,952×64 | N/A | 64 |
| Batch Normalization | N/A | N/A | Relu | 64 |
| Fully connected | 16 | 64×16 | N/A | 16 |
| Output layer | N/A | 16×1 | Sigmoid | 1 |

^a^N/A: not applicable.

^b^ReLu: rectified linear unit.
